# Invasive pneumococcal disease surveillance in Canada, 2023

**DOI:** 10.14745/ccdr.v52i0102a05

**Published:** 2026-02-19

**Authors:** Alyssa Golden, Averil Griffith, Brigitte Lefebvre, Allison McGeer, Gregory Tyrrell, Julianne Kus, Jennifer Grant, Jessica Minion, Paul Van Caeseele, Guillaume Desnoyers, David Haldane, Yang Yu, Xiaofeng Ding, Laura Steven, Jan McFadzen, George Zhanel, Courtney Primeau, Irene Martin

**Affiliations:** 1 National Microbiology Laboratory, Public Health Agency of Canada, Winnipeg, MB; 2 Laboratoire de santé publique du Québec, Institut national de santé publique du Québec, Sainte-Anne-de- Bellevue, QC; 3 Toronto Invasive Bacterial Diseases Network (TIBDN), Department of Microbiology, Mount Sinai Hospital, Toronto, ON; 4 Provincial Laboratory for Public Health, Edmonton, AB; 5 Public Health Ontario, Toronto, ON; 6 Department of Laboratory Medicine and Pathobiology, University of Toronto, Toronto, ON; 7 British Columbia Centre for Disease Control, Vancouver, BC; 8 Roy Romanow Provincial Laboratory, Regina, SK; 9 Cadham Provincial Laboratory, Winnipeg, MB; 10 Laboratoire de santé publique du New Brunswick, Moncton, NB; 11 Queen Elizabeth II Health Science Centre, Halifax, NS; 12 Newfoundland and Labrador Public Health Laboratory, St. John’s, NL; 13 Queen Elizabeth Hospital, Charlottetown, PE; 14 Stanton Territorial Hospital, Yellowknife, NT; 15 Yukon Communicable Disease Control, Whitehorse, YK; 16 Department of Medical Microbiology and Infectious Diseases, Max Rady College of Medicine, Rady Faculty of Health Sciences, University of Manitoba, Winnipeg, MB; 17 Centre for Emerging and Respiratory Infections and Pandemic Preparedness, Public Health Agency of Canada, Ottawa, ON

**Keywords:** IPD, Canada, *Streptococcus pneumoniae*, PCV15, PCV20, V116, pneumococcus, serotype, surveillance, antimicrobial resistance

## Abstract

**Background:**

Invasive pneumococcal disease (IPD), which is caused by *Streptococcus pneumoniae*, has been a nationally notifiable disease in Canada since 2000. This report summarizes the demographics, serotypes and antimicrobial resistance of IPD isolates collected in Canada in 2023.

**Methods:**

The Public Health Agency of Canada’s National Microbiology Laboratory (Winnipeg, Manitoba) collaborates with provincial and territorial public health laboratories to conduct national surveillance of IPD. Serotypes and minimum inhibitory concentrations were obtained from whole genome sequencing data.

**Results:**

The incidence of IPD in Canada was 10.2 cases per 100,000 population in 2022, increasing from the low rate of 5.6 cases per 100,000 population in 2021. A total of 4,760 IPD isolates were collected during 2023. The most common serotypes overall were 3 (12.3%, n=587), 4 (12.2%, n=580), 22F (8.2%, n=391) and 9V (7.1%, n=340). Serotypes 4 (7.1%−12.2%, *p*<0.0001) and 9V (1.3%−7.1%, *p*<0.0001) also increased significantly in prevalence since 2019, resulting in an overall increase in the proportion of PCV15 and PCV20/non-V116 serotypes causing disease. The highest rates of antimicrobial resistance in 2023 were seen with clarithromycin (25.8%), doxycycline (17.5%) and trimethoprim/sulfamethoxazole (15.9%). Multidrug-resistance continued to increase from 2019 to 2023 (8.4%–13.2%, *p*<0.0001) and rates were highest in serotypes 9V, 15A, 19A, 23A and 35B.

**Conclusion:**

The high number of IPD cases collected in 2023 represents a return to pre-SARS-CoV-2 pandemic disease activity. Several serotypes included in previous conjugate vaccine formulations are highly common or are increased in prevalence, including 3, 4 and 9V. Continued surveillance of pneumococcal serotypes is imperative to evaluate vaccine effectiveness, particularly as new vaccine formulations are approved and integrated into immunization schedules in Canada.

## Introduction

*Streptococcus pneumoniae* is a common gram-positive microorganism that can cause severe invasive pneumococcal diseases (IPDs), such as bacteremia and meningitis. Children, the elderly and immunocompromised individuals are at greatest risk (1). Pneumococcal conjugate vaccines (PCVs) have been used with great success, decreasing the burden of IPD worldwide. Recently, PCVs with expanded coverage were approved for use in Canada, including 15-valent (PCV15: 1, 3, 4, 5, 6A, 6B, 7F, 9V, 14, 18C, 19A, 19F, 22F, 23F, and 33F), 20-valent (PCV20: PCV15 serotypes plus 8, 10A, 11A, 12F and 15BC) and 21-valent (V116: 3, 6A, 7F, 8, 9N, 10A, 11A, 12F, 15A, 15BC, 16F, 17F, 19A, 20A, 22F, 23A, 23B, 24F, 31, 33F and 35B) formulations. While PCV15 and PCV20 have followed the pattern of adding additional serotypes to a previous formulation, V116 is the first PCV specifically designed for use in adults over 18 years of age, containing a series of unique serotypes commonly responsible for adult pneumococcal disease (2). As of the time of writing, PCV15 and PCV20 are the pneumococcal vaccines recommended by Canada’s National Advisory Committee on Immunization for routine paediatric immunization, while PCV20 and V116 are recommended for adults over 65 years of age (3–5). The objective of this annual surveillance report is to provide a summary of the serotypes and antimicrobial resistance associated with IPD in Canada in 2023.

## Methods

### Surveillance program

As previously described, surveillance of IPD in Canada consists of a passive, laboratory-based system where invasive isolates from the provincial and territorial public health laboratories are sent to the National Microbiology Laboratory (NML, Winnipeg), Alberta Provincial Laboratory for Public Health or *Laboratoire de santé publique du Québec* for serotyping ((6)). In 2023, a total of 4,760 IPD isolates were reported, including 2,932 submitted directly to NML by provincial and territorial public health laboratories and data for a further 824 and 1,004 isolates collected and tested by Alberta Provincial Laboratory for Public Health and *Laboratoire de santé publique du Québec*, respectively (**Table 1**). Sterile clinical isolation sites include blood, cerebrospinal fluid, peritoneal, pericardial or joint fluid, internal body sites and deep tissue including surgical or biopsy samples. For this report, isolates from pleural fluid (empyema) are included, despite not meeting the current national case definition for invasive disease, as they are considered invasive in some jurisdictions (7,8). Note that this analysis does not include typing data for PCR-positive samples with no culture data.

**Table 1:** Number of invasive *Streptococcus pneumoniae* submitted by province, 2023

Province	Age group (years)						Not given	Total
	Younger than 2	2–4	5–14	15–49	50–64	65 and older		
British Columbia^a^	3	9	6	195	196	199	3	611
Alberta	16	15	18	317	230	207	21	824
Saskatchewan	5	3	5	146	73	85	0	317
Manitoba	7	4	13	136	86	81	1	328
Ontario	47	61	48	312	428	587	2	1,485
Québec	53	40	29	175	245	450	12	1,004
Atlantic^b^	4	6	5	23	54	77	4	173
Northern^c^	0	1	0	5	4	8	0	18
Total	135	139	124	1,309	1,316	1,694	43	4,760

^a^ Includes isolates from Yukon

^b^ Includes isolates from New Brunswick, Prince Edward Island, Nova Scotia and Newfoundland and Labrador

^c^ Includes isolates from Northwest Territories and Nunavut

Population-based incidence of IPD up to 2022 were obtained through the Canadian Notifiable Disease Surveillance System (CNDSS). Incidence rates for 2023 were not available at the time of writing. Population data for incidence rates were obtained from Statistics Canada’s July 1^st^ annual population estimates.

### Isolate testing

All IPD isolates submitted to NML in 2023 were tested by whole-genome sequencing (WGS) using the Illumina platform. Isolates were confirmed as *S. pneumoniae* using *rpoB* sequence analysis (9). Serotypes were identified directly using the WADE pipeline (https://github.com/phac-nml/wade). Isolates that were nontypeable by WGS were confirmed by Quellung reaction, using commercial antisera (SSI Diagnostica; Statens Serum Institut, Copenhagen, Denmark) (10). Serotyping of IPD at Alberta Provincial Laboratory for Public Health and *Laboratoire de santé publique du Québec* was performed by the Quellung reaction. For this study, serotypes 15B and 15C were grouped together as 15BC because of reported reversible switching between them *in vivo* during infection, making it difficult to differentiate between the two types (11,12).

For all isolates submitted to NML, minimum inhibitory concentrations were predicted using WADE and previously described algorithms for penicillin, ceftriaxone, chloramphenicol, clarithromycin, clindamycin, doxycycline, levofloxacin and trimethoprim/sulfamethoxazole (13). Minimum inhibitory concentrations were interpreted using Clinical and Laboratory Standards Institute breakpoints (14); penicillin was interpreted using the oral penicillin V breakpoints, and ceftriaxone using the meningitis breakpoints. Multidrug-resistance (MDR) was defined as resistance to three or more different classes of antimicrobials. Genomes were scanned for *van* family genes to identify any emergence of vancomycin-nonsusceptibility (15).

### Data analysis

Demographic data submitted with bacterial isolates included patient age, sex, clinical source, province and date of collection. Duplicate isolates collected from the same patient within 21 days were counted once if they were the same serotype, with the most invasive isolation site assigned. Meningitis related isolates were regarded as most invasive, followed by blood and then other sterile sites. Data was aggregated by age into younger than two, 2–4, 5–14, 15–49, 50–64 and 65 years and older age groups and regionally into Western (British Columbia, Alberta, Saskatchewan, Manitoba), Central (Ontario and Québec), Eastern (New Brunswick, Nova Scotia, Prince Edward Island, Newfoundland and Labrador) and Northern (Yukon Territories, Northwest Territories and Nunavut) regions of Canada. Statistical significance of trends was assessed using the Cochran-Armitage test of trend, with a *p*-value of <0.05 considered significant.

## Results

**Figure 1:**
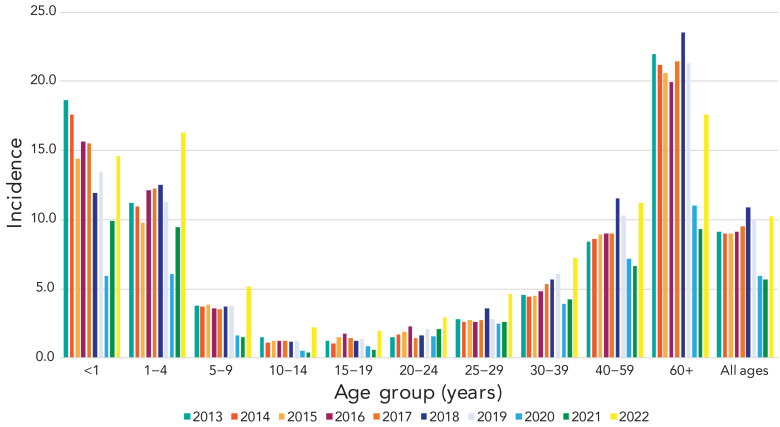
Annual incidence of invasive pneumococcal disease cases per 100,000 population in Canada by age group, 2013–2022^a^ ^a^ Data from Canadian Notifiable Disease Surveillance System

The overall incidence of IPD in Canada increased to 10.2 cases per 100,000 population in 2022. This represents a sharp increase from the 2021 incidence of 5.6 cases per 100,000 population, and a return to pre-COVID-19 pandemic incidence that peaked in 2018 at 10.9 cases per 100,000 population (**Figure 1**). There was a large increase in the number of IPD isolates submitted in 2023 (n=4,760) in comparison to 2022 (n=3,867), representing the highest annual IPD isolate total collected by NML to-date.

Of the 4,760 IPD isolates tested in 2023, 4,717 (99.1%) had patient ages. Infants younger than two years of age accounted for 2.9% (n=135), toddlers aged 2–4 years for 2.9% (n=139), children aged 5–14 years for 2.6% (n=124), patients aged 15–49 years for 27.8% (n=1,309), older adults aged 50–64 years for 27.9% (n=1,316) and seniors aged 65 years and older for 35.9% (n=1,694). Forty-three isolates had no ages provided. Of the isolates for which sex information was available, isolates from male patients represented 57.0% (n=2,653) of isolates. Blood was the main clinical isolation site, accounting for 92.8% (n=4,418) of isolates collected.

**Figure 2:**
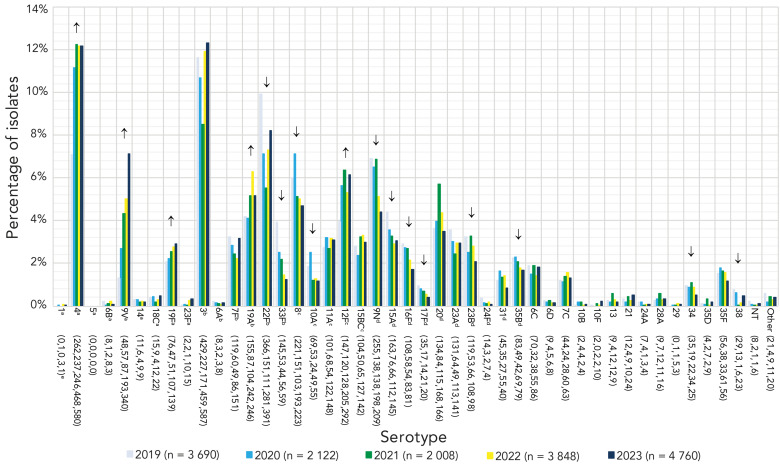
Invasive *Streptococcus pneumoniae* serotype prevalence trends, all age groups combined, 2019–2023^a,b,c,d,e^ ^a^ PCV15 and PCV20/non-V116 serotype ^b^ PCV15, PCV20 and V116 serotype ^c^ PCV20 and V116 serotype ^d^ V116/non-PCV15/20 serotype ^e^ Number of isolates for 2019, 2020, 2021, 2022 and 2023, respectively. For serotypes with an overall (2019–2023) N≥50: up or down arrows indicate statistically significant trends toward increasing or decreasing prevalence for the 2019–2023 timespan, using the chi-squared test for trend. Serotypes with no arrow either did not demonstrate a statistically significant trend, or did not have an overall N≥50. Serotypes 15B and 15C were grouped together as 15B/C because of reported reversible switching between them *in vivo* during infection, making it difficult to precisely differentiate between the two types (11,12)

The most commonly collected serotypes overall in 2023 were 3 (12.3%, n=587), 4 (12.2%, n=580), 22F (8.2%, n=391) and 9V (7.1%, n=340). Other common types included 12F, 19A, 8 and 9N. Serotypes that demonstrated significant increasing trends in prevalence from 2019 to 2023 include PCV15 and PCV20/non-V116 serotypes 4 (7.1%−12.2%, *p*<0.0001), 9V (1.3%−7.1%, *p*<0.0001) and 19F (2.1%−2.9%, *p*=0.0051); PCV15/PCV20/V116 serotype 19A (4.2%–5.2%, *p*=0.0011); and PCV20/V116 serotype 12F (4.0%–6.1%, *p*=0.0002) (**Figure 2**). Vaccine serotypes that significantly decreased in prevalence from 2019 to 2023 included PCV15/PCV20/V116 serotypes 22F and 33F (*p*≤0.018), PCV20/V116 serotypes 8 and 10A (*p*≤0.0002) and V116-unique serotypes 9N, 15A, 16F, 17F, 23B, 35B (*p*≤0.0196) (Figure 2).

The most common serotypes in children younger than two years during 2023 were 15BC (17.8%, n=24) and 19A (16.3%, n=22). For children aged 2–4 years and 5–14 years, serotypes 19A (19.4%, n=27; 16.1%, n=20, respectively) and 3 (15.1%, n=21; 18.5%, n=23) were most common. For patients aged 15–49 years, serotype 4 was the most prevalent (22.6%, n=296), followed by serotypes 9V (10.8%, n=141), 12F (10.0%, n=131) and 3 (9.8%, n=128). Serotypes 4 (13.3%, n=175), 3 (12.8%, n=169) and 9V (8.8%, n=116) were the most common in adults 50–64 years, while serotypes 3 (14.1%, n=239) and 22F (11.7%, n=198) were dominant in adults over 65 years of age.

**Figure 3:**
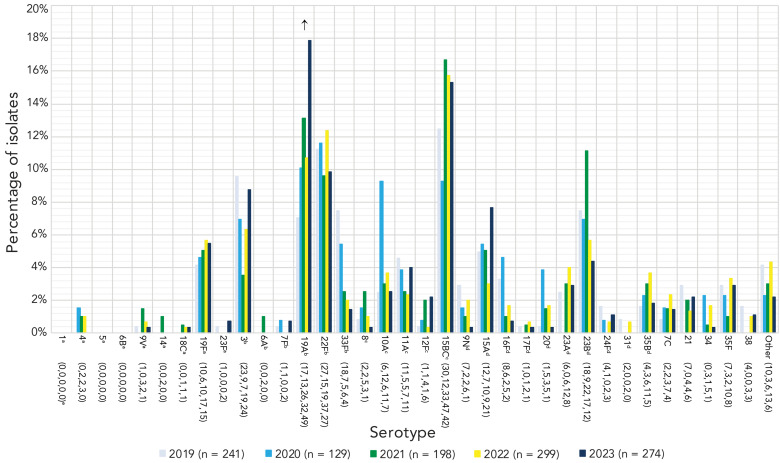
Invasive *Streptococcus pneumoniae* serotype prevalence trends, younger than five years of age, 2019–2023^a,b,c,d,e^ ^a^ PCV15 and PCV20/non-V116 serotype ^b^ PCV15, PCV20 and V116 serotype ^c^ PCV20 and V116 serotype ^d^ V116/non-PCV15/20 serotype ^e^ Number of isolates for 2019, 2020, 2021, 2022 and 2023, respectively. For serotypes with an overall (2019–2023) N≥50: up or down arrows indicate statistically significant trends toward increasing or decreasing prevalence for the 2019–2023 timespan, using the chi-squared test for trend. Serotypes with no arrow either did not demonstrate a statistically significant trend, or did not have an overall N≥50. Serotypes 15B and 15C were grouped together as 15B/C because of reported reversible switching between them *in vivo* during infection, making it difficult to precisely differentiate between the two types (11,12)

**Figure 4:**
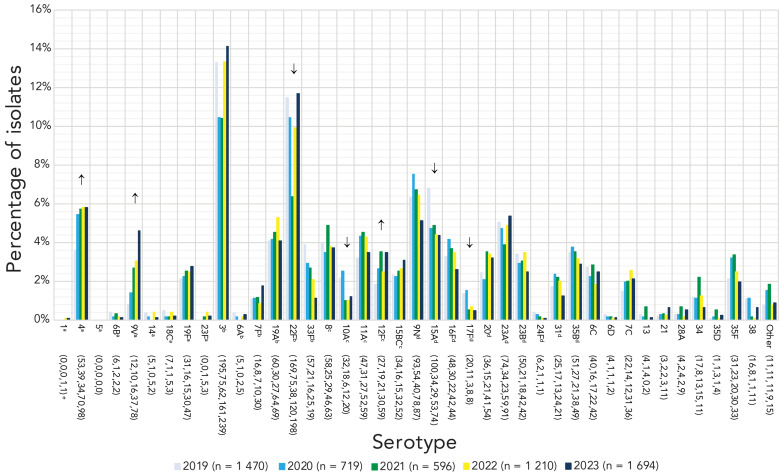
Invasive *Streptococcus pneumoniae* serotype prevalence trends, 65 years of age and older, 2019–2023^a,b,c,d,e^ ^a^ PCV15 and PCV20/non-V116 serotype ^b^ PCV15, PCV20 and V116 serotype ^c^ PCV20 and V116 serotype ^d^ V116/non-PCV15/20 serotype ^e^ Number of isolates for 2019, 2020, 2021, 2022 and 2023, respectively. For serotypes with an overall (2019–2023) N≥50: up or down arrows indicate statistically significant trends toward increasing or decreasing prevalence for the 2019–2023 timespan, using the chi-squared test for trend. Serotypes with no arrow either did not demonstrate a statistically significant trend, or did not have an overall N≥50. Serotypes 15B and 15C were grouped together as 15B/C because of reported reversible switching between them *in vivo* during infection, making it difficult to precisely differentiate between the two types (11,12)

A significant increase of serotype 19A (7.1%–17.9%, *p*=0.0007) was observed in children younger than five years of age from 2019 to 2023 (**Figure 3**), however no significant changes were noted for children five to 14 years. Patients aged 15–49 years and 50–64 years experienced large, significant increases of serotypes 4 (14.5%–22.6%, *p*<0.0001; 8.4%–13.3%, *p*<0.0001, respectively) and 9V (2.2%–10.8%, *p*<0.0001; 1.6%–8.8%, *p*<0.0001). Adults 65 years and older also experienced increases of serotypes 4 and 9V, but to a lesser extent (3.6%–5.8%, *p*=0.0064; 0.8%–4.6%, *p*=0.0093, respectively) (**Figure 4**). Patients 15–49 years of age also saw a small but significant increase in serotype 11A (1.3%–2.8%, *p*=0.0098), while adults 50–64 years saw an increase in serotype 19F (1.0%–2.7%, *p*=0.0033). Adults 50–64 years and 65 years of age and older both saw an increase of serotype 12F (4.6%–7.1%, *p*=0.0298; 1.8%–3.5%, *p*=0.0121, respectively).

The most common serotypes isolated in Western Canada in 2023 were 4 (17.4%, n=362), 3 (11.3%, n=235) and 9V (10.5%, n=217). In Central Canada, serotype 3 remained most prevalent (13.1%, n=325), followed by 22F (9.4%, n=234) and 19A (8.2%, n=205). In Eastern Canada, serotypes 3 (14.5%, n=25), 22F (11.6%, n=20) and 4 (11.0%, n=19) were the most common in 2023. Serotypes 20 (22.7%, n=5) and 9V (18.2%, n=4) were most common in Northern Canada, though submissions from this region remained low (**Appendix**, **Figure S1**).

**Figure 5:**
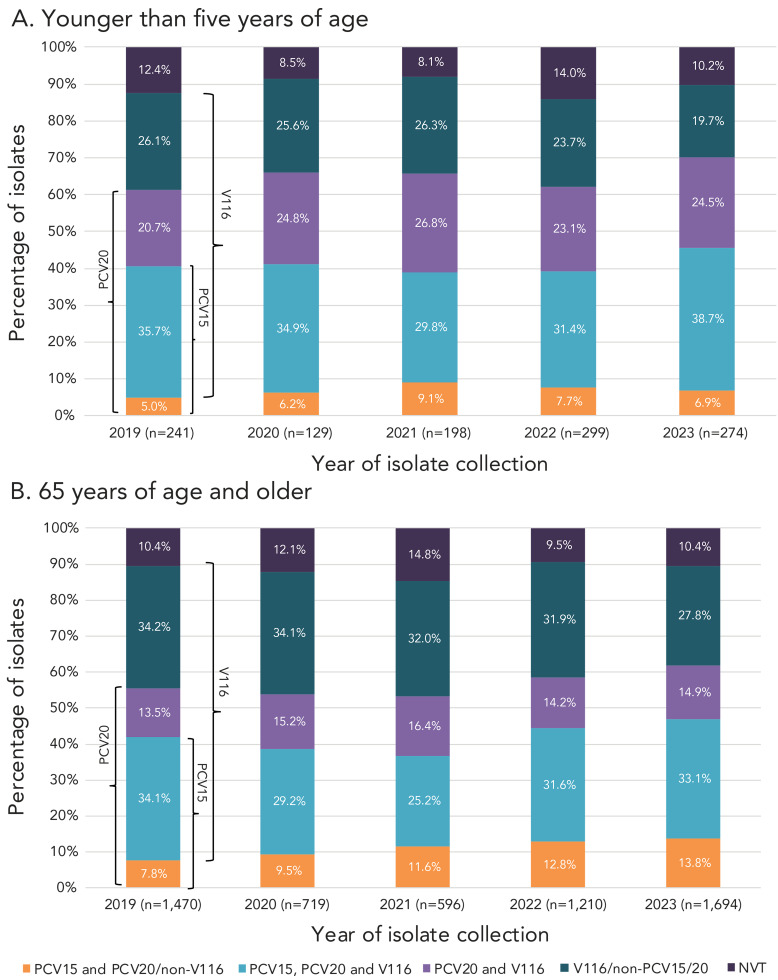
Invasive *Streptococcus pneumoniae* serotype trends by vaccine and age^a^, 2019–2023 Abbreviations: NVT, non-vaccine serotype; PCV, pneumococcal conjugate vaccine ^a^ Antimicrobial classes include: β-lactams (penicillin using the Clinical & Laboratory Standards Institute (CLSI) oral penicillin V interpretive criteria, ceftriaxone using the CLSI parenteral meningitis interpretive criteria); macrolides (clarithromycin); fluoroquinolones (levofloxacin); tetracyclines (doxycycline); folate pathway inhibitors (trimethoprim-sulfamethoxazole); phenicols (chloramphenicol); lincosamides (clindamycin)

Serotypes belonging to the currently recommended PCV15 and PCV20 vaccines, but not V116 (1, 4, 5, 6B, 9V, 14, 18C, 19F and 23F) have significantly increased in prevalence overall from 2019 to 2023 (11.4%−23.3%, *p*<0.0001); this increase was noted in all adult age groups. The proportion of serotypes included in all three formulations (3, 6A, 7F, 19A, 22F and 33F) increased in children 5–14 years of age (35.7%–50.8%, *p*=0.186), but decreased in patients 15–49 years (32.2%–24.1%, *p*=0.0007). The category of serotypes shared by PCV20 and V116 (8, 10A, 11A, 12F and 15BC) remained stable over time. The proportion of V116-unique serotypes (9N, 15A, 16F, 17F, 20, 23A, 23B, 24F, 31 and 35B) decreased significantly overall (29.5%–20.7%, *p*<0.0001); this decrease was seen in all age groups except children younger than five years, where no significant changes were identified for any vaccine category. Overall, the proportion of non-vaccine serotypes has not significantly changed (**Figure 5**; Appendix, **Table S1**).

Whole-genome sequencing-based prediction of antimicrobial susceptibilities was performed on 3,058 *S. pneumoniae* isolates collected in 2023 (**Table 2**). The highest rate of resistance during 2023 was observed for clarithromycin at 25.8% (n=790) but has remained stable from 2019 to 2023. Penicillin resistance increased over the 2019 to 2023 period, from 2.9% (n=60) to 8.0% (n=246; *p*<0.0001). Other antimicrobial resistance rates for 2023 included doxycycline at 17.5% (n=535), trimethoprim-sulfamethoxazole at 15.9% (n=486), clindamycin at 7.9% (n=242) and chloramphenicol at 2.7% (n=83). There was limited resistance to ceftriaxone (0.2%, n=5), and all isolates were susceptible to levofloxacin. No vancomycin resistance determinants were identified. Resistance rates for specific serotypes in 2023 are listed in **Table 3**.

**Table 2:** Proportion of antimicrobial resistant invasive *Streptococcus pneumoniae* isolates by year, 2019–2023

Antimicrobial	Year, n (%)				
	2019	2020	2021	2022	2023
PEN	60 (2.9)	46 (3.9)	58 (5.1)	90 (8.3)	246 (8.0)
CRO	6 (0.3)	6 (0.5)	6 (0.5)	2 (0.2)	5 (0.2)
CHL	60 (2.9)	46 (3.9)	40 (3.5)	37 (3.4)	83 (2.7)
CLR	517 (24.7)	271 (22.9)	245 (21.4)	273 (25.2)	790 (25.8)
CLI	166 (7.9)	90 (7.6)	101 (8.8)	100 (9.2)	242 (7.9)
DOX	237 (11.3)	143 (12.1)	163 (14.2)	186 (17.2)	535 (17.5)
LEV	9 (0.4)	1 (0.1)	0 (0.0)	2 (0.2)	0 (0.0)
SXT	127 (6.1)	99 (8.4)	122 (10.6)	164 (15.1)	486 (15.9)
Total tested	2,093	1,182	1,147	1,083	3,058

Abbreviations: CHL, chloramphenicol; CLI, clindamycin; CLR, clarithromycin; CRO, ceftriaxone using the Clinical & Laboratory Standards Institute parenteral meningitis interpretive criteria; DOX, doxycycline; LEV, levofloxacin; PEN, penicillin using the Clinical & Laboratory Standards Institute oral penicillin V interpretive criteria; SXT, trimethoprim/sulfamethoxazole

**Table 3:** Percentage of antimicrobial resistance among invasive *Streptococcus pneumoniae* serotypes collected in 2023

Serotype^a^	Percentage of isolates with antimicrobial resistance^b^								
	PEN	CRO	CHL	CLR	CLI	DOX	LEV	SXT	MDR
1^c^ (n=1)	-	-	-	-	-	-	-	100	-
4^c^ (n=354)	-	-	2.0	5.1	4.2	8.2	-	19.8	4.2
9V^c^ (n=204)	64.7	1.0	0.5	67.2	-	66.7	-	69.1	67.2
14^c^ (n=9)	88.9	-	-	88.9	77.8	66.7	-	88.9	77.8
18C^c^ (n=8)	-	-	-	37.5	-	37.5	-	37.5	37.5
19F^c^ (n=98)	5.1	-	-	6.1	6.1	5.1	-	1.0	5.1
23F^c^ (n=15)	-	-	-	26.7	20.0	20.0	-	20.0	13.3
3^d^ (n=351)	0.3	-	9.4	10.8	8.5	12.0	-	0.9	8.3
6A^d^ (n=8)	12.5	-	-	75.0	-	-	-	-	-
7F^d^ (n=116)	-	-	-	15.5	15.5	15.5	-	0.9	15.5
19A^d^ (n=112)	24.1	1.8	3.6	51.8	41.1	27.7	-	25.0	30.4
22F^d^ (n=234)	-	-	2.1	51.3	2.1	2.6	-	1.3	2.1
33F^d^ (n=42)	4.8	-	-	90.5	-	-	-	19.0	-
8^e^ (n=143)	-	-	0.7	1.4	-	2.1	-	-	-
10A^e^ (n=29)	-	-	-	6.9	-	-	-	-	-
11A^e^ (n=97)	-	-	-	25.8	4.1	4.1	-	14.4	3.1
12F^e^ (n=235)	-	-	3.4	39.6	0.9	41.7	-	41.3	3.8
15BC^e,f^ (n=113)	4.4	0.9	-	26.5	5.3	8.8	-	5.3	6.2
9N^g^ (n=130)	2.3	-	-	12.3	3.1	10.8	-	4.6	7.7
15A^g^ (n=82)	13.4	-	1.2	51.2	42.7	43.9	-	2.4	43.9
16F^g^ (n=51)	-	-	3.9	9.8	7.8	7.8	-	2.0	7.8
17F^g^ (n=17)	-	-	-	11.8	5.9	5.9	-	-	5.9
20^g^ (n=123)	-	-	-	4.1	4.1	4.1	-	0.8	4.1
23A^g^ (n=87)	1.1	-	-	26.4	26.4	26.4	-	2.3	26.4
23B^g^ (n=65)	1.5	-	1.5	7.7	4.6	3.1	-	10.8	1.5
24F^g^ (n=3)	-	-	-	100	100	100	-	-	100
31^g^ (n=26)	-	-	-	3.8	-	-	-	-	-
35B^g^ (n=57)	66.7	-	1.8	59.6	3.5	3.5	-	21.1	21.1
6C (n=44)	6.8	-	6.8	29.5	4.5	13.6	-	27.3	15.9
6D (n=8)	-	-	87.5	12.5	12.5	75.0	-	87.5	75.0
7A (n=1)	-	-	-	100	100	100	-	-	100
7C (n=39)	-	-	2.6	7.7	7.7	7.7	-	64.1	7.7
9A (n=1)	100	-	-	100	-	100	-	100	100
10B (n=3)	-	-	33.3	33.3	33.3	100	-	33.3	33.3
12A (n=2)	-	-	-	-	-	-	-	50.0	-
13 (n=7)	-	-	-	85.7	85.7	85.7	-	42.9	85.7
17A (n=1)	-	-	-	100	100	100	-	-	100
21 (n=22)	-	-	-	-	-	-	-	13.6	-
24A (n=4)	-	-	-	25.0	-	-	-	75.0	-
28A (n=12)	-	-	58.3	-	-	58.3	-	-	-
34 (n=23)	-	-	-	13.0	13.0	26.1	-	30.4	13.0
35A (n=2)	-	-	-	100	-	100	-	100	100
35D (n=9)	55.6	-	-	55.6	-	11.1	-	11.1	22.2
35F (n=39)	-	-	-	10.3	2.6	-	-	-	-
38 (n=16)	-	-	-	12.5	-	25.0	-	6.3	-
NT (n=4)	50.0	-	-	100	25.0	100	-	25.0	50.0

Abbreviations: CHL, chloramphenicol; CLI, clindamycin; CLR, clarithromycin; CRO, ceftriaxone using the Clinical & Laboratory Standards Institute parenteral meningitis interpretive criteria; DOX, doxycycline; LEV Levofloxacin; PEN, penicillin using the Clinical & Laboratory Standards Institute oral penicillin V interpretive criteria; SXT, trimethoprim/sulfamethoxazole

^a^ The following serotypes were fully antimicrobial susceptible in 2023: 10F (n=3), 11B (n=1), 18A (n=1), 22A (n=1), 24B (n=1), 28F (n=1), 37 (n=3)

^b^ “-“ denotes no resistance (0%) to the antimicrobial

^c^ PCV15 and PCV20/non-V116 serotype

^d^ PCV15, PCV20 and V116 serotype

^e^ PCV20 and V116 serotype

^f^ Serotypes 15B and 15C were grouped together as 15B/C because of reported reversible switching between them *in vivo* during infection, making it difficult to precisely differentiate between the two types (11,12)

^g^ V116/non-PCV15/20 serotype

**Figure 6:**
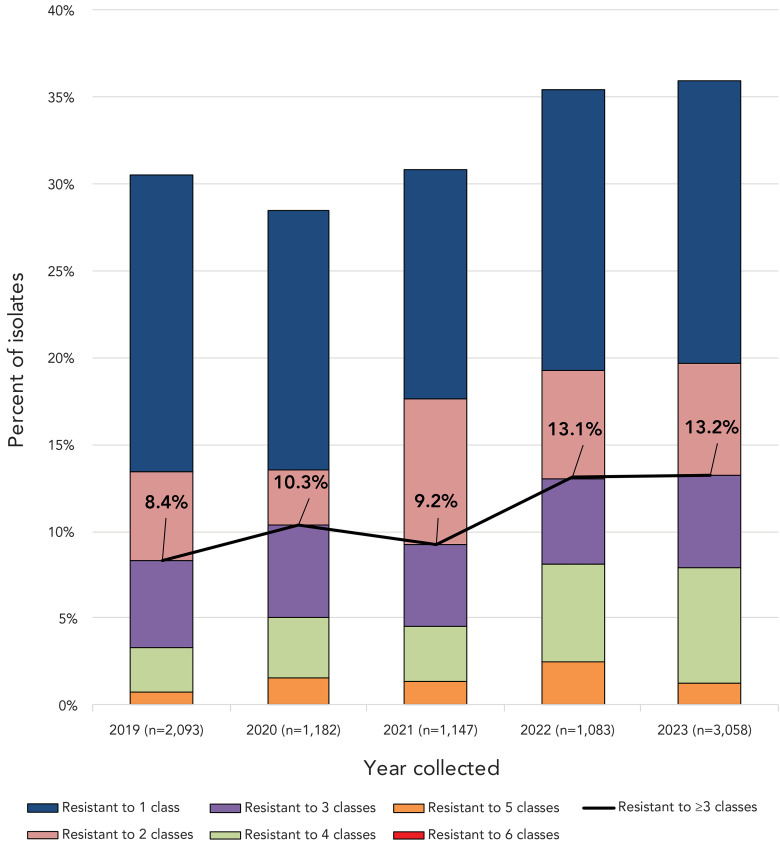
Annual trend of multidrug resistance of invasive *Streptococcus pneumoniae*, 2019–2023^a^ ^a^ Antimicrobial classes include: β-lactams (penicillin using the Clinical & Laboratory Standards Institute (CLSI) oral penicillin V interpretive criteria, ceftriaxone using the CLSI parenteral meningitis interpretive criteria); macrolides (clarithromycin); fluoroquinolones (levofloxacin); tetracyclines (doxycycline); folate pathway inhibitors (trimethoprim-sulfamethoxazole); phenicols (chloramphenicol); lincosamides (clindamycin)

Multidrug-resistant IPD increased from 8.4% (n=175) of the isolates tested in 2019 to 13.2% (n=404) in 2023 (*p*<0.0001) (**Figure 6**). Of the serotypes where 10 or more isolates were collected in 2023, the highest rates of MDR were identified in 9V (67.2%, n=137), 15A (43.9%, n=36), 19A (30.4%, n=35), 23A (26.4%, n=23) and 35B (21.1%, n=12) (Table 3; Appendix, **Figure S2**). Together these serotypes accounted for 59.9% (n=242/404) of MDR isolates collected in 2023. The most common MDR pattern in 2023 was β-lactam-macrolide-tetracycline-trimethoprim/sulfamethoxazole, with serotype 9V accounting for the majority (n=130). Serotypes 15A and 23A both had macrolide-clindamycin-tetracycline as their most common MDR pattern (n=23 and n=22, respectively). Multidrug-resistant serotype 19A isolates were most commonly resistant to five antimicrobial classes (β-lactam, macrolide, clindamycin, tetracycline and trimethoprim/sulfamethoxazole; n=23), while the most common MDR pattern for serotype 35B was β-lactam-macrolide-trimethoprim/sulfamethoxazole (n=10) (Appendix, **Table S2**).

## Discussion

The COVID-19 pandemic and subsequent containment measures had a significant impact on the spread of respiratory pathogens such as *S. pneumoniae* (16). In 2020 and 2021, IPD incidence in Canada was the lowest seen since 2001, at 5.89 and 5.63 cases per 100,000, respectively. In 2022, 3,984 cases of IPD were reported to CNDSS, with a national incidence rate of 10.2 cases per 100,000 population. This represents a return to pre-pandemic incidence, where rates had gradually increased to a high of 10.86 cases per 100,000 population in 2018. Though 2023 incidence was not available at the time of publication, the sharp increase in the number of isolates submitted for testing in 2023 (n=4,760) in comparison to the previous highest annual count reported to CNDSS in 2018 (n=4,026) suggests that 2023 incidence was higher than 2022. This situation is not unique to Canada; reports from other countries have also indicated a return to pre-pandemic disease activity in 2023 (17–19), including some where rates have now surpassed pre-pandemic years (20,21).

Serotype 3, a PCV13 vaccine serotype, was the most common type collected in Canada in 2023, edging out serotype 4 by a very small margin. With the exception of 2021, prevalence of serotype 3 has been stable across Canada during the study period, and particularly high among adult age groups. Annual reports from Hong Kong, Spain and Denmark list serotype 3 as the most common serotype in 2023 (17,19,20), with Ireland, Belgium and New Zealand placing it in their top three most common types (18,21,22). Other vaccine serotypes were also common in Canada during 2023, particularly serotypes 4 and 9V, which have been included in conjugate vaccines since the original PCV-7 formulation. Serotype 4 has been increasing in Canada for a number of years (6,23). In 2023 it was particularly common in Western Canada, and the most common type collected from adults aged 15–49 years and 50–64 years. The most recent annual report from Belgium described a similar distribution, with serotype 4 as the third most common type overall, but the most common type in adults 16–49 years of age (18). A recent study in the United States has identified that risk of invasive disease is highest at the time of first acquisition, noting that serotype 4 has a particularly low duration of carriage which allows it to transition quickly to disease in susceptible populations (24). Thus, Beall *et al.* have postulated that serotype 4 is rapidly transmitted directly between adults, causing IPD shortly after initial acquisition (25). This supports the results of previous studies in Western North America that have associated serotype 4 infections with susceptible adult populations with risk factors such as homelessness and substance use (26,27).

Serotype 9V has not been described as readily in the literature as other vaccine types and was not mentioned in the 2023 annual reports of other countries published at the time of writing this manuscript (18,19,21,22). In our study, serotype 9V was the second most common invasive serotype in adults 15–49 years, the third most common type in adults 50–64 years and the third most common type collected from Western Canada. Prevalence of serotype 9V has consistently increased in Canada over the course of the pandemic; in contrast, minimal numbers of 9V isolates were collected by the 30 participating countries comprising the Invasive Respiratory Infections Surveillance Consortium, with an overall decrease in prevalence going into the pandemic years (16). Based on the comparable age distributions, it is possible that serotype 9V is similar to serotype 4 in that it is associated with certain risk factors that are less common in children and older adults (e.g., substance use). Epidemiological data is required to confirm this theory. Serotypes 4 and 9V are also similar in that neither is included in the novel, adult-specific V116 vaccine. While advisory boards such as the United States Advisory Committee on Immunization Practices have recommended this vaccine for use in adults for whom PCV use is indicated, the Advisory Committee on Immunization Practices has also noted that this vaccine may not be an appropriate choice in jurisdictions where serotype 4 is highly prevalent in adults (28). In Canada, this logic may be applied similarly for both serotype 4 and 9V.

Serotype 9V was one of the most highly antimicrobial-resistant serotypes in 2023, with high rates of resistance to β-lactams, macrolides, tetracyclines and trimethoprim/sulfamethoxazole. Penicillin-resistant serotype 9V was among the initially described, widely disseminated antimicrobial-resistant clones defined by the Pneumococcal Molecular Epidemiology Network in 2001 (29); inclusion of this type in the first conjugate vaccine (PCV-7) decreased the incidence of IPD caused by this resistant type (7). The return of serotype 9V has also been associated with substantial MDR; 67.2% of 9V isolates were MDR, which accounted for 33.9% of the total MDR isolates collected in 2023. The inclusion of this serotype in PCV20 (but not V116) suggests it may be a more appropriate choice for both paediatric and adult immunization in jurisdictions where serotype 9V is highly prevalent. However, V116 does cover three of the five most common MDR serotypes in Canada that are not included in PCV20 (15A, 23A, 35B) in addition to 19A, suggesting that use of these vaccines in tandem could potentially prevent a substantial number of antimicrobial-resistant infections in Canada.

As Canada reaches a point where multiple PCV formulations are equally recommended for use, it will be important to monitor how the serotype distribution adjusts, both nationally and regionally, along with antimicrobial resistance rates. With provinces and territories free to choose which vaccines to offer based on procurement and epidemiological considerations, serotype distribution may vary by jurisdiction more than ever before.

## Limitations

Caution should be exercised when interpreting the data presented in this report. Provinces and territories may only submit a subset of their isolates to NML for testing. Numbers of isolates submitted to NML versus information submitted to CNDSS, may differ due to differences in submission protocols from the provinces. Data for 2020 and 2021 may not be reflective of actual trends, as the COVID-19 pandemic impacted disease incidence in all age groups.

## Conclusion

Incidence of IPD in Canada in 2022 increased considerably following the COVID-19 pandemic, and the high number of IPD cases collected in 2023 represents a return to pre-pandemic disease activity. Several serotypes included in previous conjugate vaccine formulations (PCV7, PCV13) were common in 2023 (serotype 3), including some that significantly increased in prevalence (serotypes 4, 9V). Continued surveillance of pneumococcal serotypes is imperative to evaluate vaccine effectiveness, particularly as new vaccine formulations are approved and integrated into immunization schedules in Canada.

## Appendix

Supplemental material is available upon request to the author: alyssa.golden@phac-aspc.gc.ca

Figure S1: Number of invasive *Streptococcus pneumoniae* isolates collected in 2023, by region and serotype

Table S1: Proportion of vaccine serotypes by age group, 2019–2023

Figure S2: Invasive *Streptococcus pneumoniae* serotypes by resistance to different antimicrobial classes, 2023

Table S2: Multidrug resistance profiles of invasive *Streptococcus pneumoniae* serotypes, 2023
